# Binding Point Recognition and Localization and Manipulator Binding Path Planning for a Rebar Binding Robot

**DOI:** 10.3390/s26041315

**Published:** 2026-02-18

**Authors:** Linjie Dong, Renfei Zhang, Zikang Shao, Ziqiu Bian, Xingsong Wang

**Affiliations:** School of Mechanical Engineering, Southeast University, Nanjing 211189, China; 230228047@seu.edu.cn (L.D.);

**Keywords:** rebar binding robot, binding point recognition, binding point localization, path planning

## Abstract

Rebar binding is a labor-intensive and low-efficiency process in the production of reinforced concrete prefabricated components, in which consistent binding quality is difficult to guarantee. To address the engineering challenges faced by rebar binding robots in complex construction environments—particularly in terms of binding-point recognition accuracy, real-time performance, and manipulator path planning efficiency—this paper presents an integrated method for binding-point recognition, localization, and binding path planning tailored to rebar binding tasks. First, based on the YOLOv8n-pose architecture, a lightweight rebar binding-point recognition and localization model, termed YOLOv8n-pose-Binding, is developed by introducing multi-scale Ghost convolution structures and an adaptive threshold focal loss. The proposed model improves keypoint detection accuracy and real-time performance while effectively reducing computational complexity, making it suitable for deployment on resource-constrained mobile robotic platforms. Second, a dedicated target coordinate system for rebar binding points is constructed to enable accurate pose estimation in the manipulator base frame. Furthermore, considering the non-uniform obstacle distribution in rebar mesh environments and the high-dimensional motion characteristics of robotic manipulators, systematic improvements are introduced to the RRT-Connect framework from the perspectives of sampling strategies, tree expansion, node reconnection, and path pruning, resulting in an improved RRT-Connect path planning algorithm. Simulation and experimental results demonstrate that, while maintaining favorable real-time performance, the proposed method achieves stable improvements in recognition accuracy and inference efficiency compared with the baseline YOLOv8n-pose model. In addition, the improved RRT-Connect algorithm exhibits superior engineering performance in terms of path planning efficiency and path quality, providing a deployable technical solution for automated rebar binding operations.

## 1. Introduction

Reinforced concrete prefabricated components, as an essential part of building industrialization, have been widely applied in residential construction, bridge engineering, and other fields [1,2]. Prior to the forming of prefabricated components, the binding of intersecting nodes in rebar meshes must be completed [3], which represents one of the most labor-intensive and efficiency-limited stages in the production of reinforced concrete components [4]. Although electric rebar binding tools can improve manual operation efficiency to some extent, they still rely heavily on human labor and fail to fundamentally reduce physical workload. In practical construction scenarios, workers are often required to maintain bent-over or semi-squatting postures while performing repetitive binding tasks, which not only leads to fatigue accumulation and occupational injuries [5], but also increases the likelihood of quality defects such as missed bindings or insufficient binding strength, thereby adversely affecting the forming quality and production efficiency of prefabricated components [6]. Compared with traditional manual operations, robotic systems exhibit clear advantages in terms of operational consistency, safety, and adaptability to complex environments. Consequently, replacing manual rebar binding with robotic systems has become an important direction for advancing automation and intelligence in the construction industry.

In recent years, with the development of robotic technologies, various automated and semi-automated robotic systems have emerged in the field of rebar binding. For example, Advanced Construction Robotics developed the Tybot system for automated binding of bridge rebar meshes [7]; Chiba Institute of Technology proposed the “T-iROBO Rebar” robot, which performs continuous binding of rebar intersection nodes using a mobile platform [8]; and Georgia Institute of Technology explored rebar binding operations based on a quadruped robotic platform [9]. In academic research, Jin [4] and Li [10], Feng [11] and Yang [12], as well as Tan [13] and Deng [14], have investigated rebar binding robots from the perspectives of perception, mechanism design, and path planning. Although these studies have achieved notable progress in rebar intersection recognition and binding execution, most existing systems still rely on relatively structured or controlled working environments and exhibit limitations in automation level, system robustness, motion flexibility, and adaptability to complex outdoor construction conditions, making it difficult to meet the practical requirements of continuous and efficient binding of large-scale rebar meshes.

To realize automated rebar binding operations, two key challenges must be addressed: accurate recognition and localization of rebar binding points [15], and efficient planning of manipulator binding paths. Rebar intersection recognition and localization mainly involve two aspects: determining whether a rebar intersection has already been bound to avoid redundant operations, and estimating the pose of unbound intersections to provide accurate target pose information for the binding mechanism. Existing methods can generally be categorized into line-based detection methods and keypoint-based detection methods. Line-based approaches typically employ traditional image processing techniques such as the Hough transform [16,17] and the RANSAC algorithm [18]. These methods feature simple processing pipelines and low computational cost, but are sensitive to noise and environmental variations, which limits their applicability in complex and dynamic construction scenes. In contrast, keypoint-based detection methods can directly extract structural features of rebar intersections and thus exhibit stronger robustness in complex environments. Representative approaches include the Active Shape Model (ASM) [19,20], cascaded pose regression [21], and supervised descent methods [22]. With the rapid development of deep learning techniques, numerous convolutional neural network-based object detection and pose estimation methods have been introduced into keypoint detection tasks. The R-CNN framework and its improved variants, Fast R-CNN and Faster R-CNN [23,24,25], demonstrate high detection accuracy but suffer from complex two-stage architectures and high computational overhead, which restrict their deployment on mobile robotic platforms with strict real-time requirements. To improve detection speed, single-stage detection methods have attracted increasing attention, among which the YOLO family is particularly representative [26]. Existing studies have shown that YOLO-based methods can achieve a favorable balance between detection accuracy and real-time performance in object detection and keypoint recognition tasks [27,28,29]. However, as model scale and parameter count continue to increase, YOLO-based models still encounter real-time performance bottlenecks when deployed on mobile robotic platforms with limited computational resources, highlighting the need for further reduction in computational complexity while maintaining accuracy.

In terms of manipulator path planning, existing approaches mainly include graph-search-based methods [30,31,32], sampling-based methods [33], and learning-based methods [34,35]. Graph-search-based algorithms exhibit good optimality in low-dimensional and structured environments but suffer from the curse of dimensionality in high-dimensional joint spaces. Learning-based methods can achieve high planning efficiency in specific scenarios, yet they typically require large-scale training data and still face challenges in interpretability and safety guarantees. By contrast, RRT and its variants (e.g., RRT*, Bi-RRT) demonstrate strong engineering practicality and robustness in high-dimensional continuous spaces and have become mainstream approaches for manipulator path planning. Although existing studies have improved RRT-based algorithms in terms of path quality and convergence speed [36,37], there remains room for further improvement in search efficiency in narrow spaces, path redundancy reduction, and the handling of task-specific constraints.

To address the above challenges, this paper is developed based on a newly designed tracked rebar binding robot system. A rebar binding point recognition and localization method is established, and an improved manipulator path planning algorithm is proposed within the RRT-Connect framework to enhance the overall efficiency and path quality of rebar binding operations. The main contributions of this paper are summarized as follows:

(1) Based on the YOLOv8n-pose model, a lightweight rebar binding point recognition and localization model, termed YOLOv8n-pose-Binding, is proposed through network structure optimization and loss function improvement, enabling real-time deployment on mobile robotic platforms with limited computing resources while maintaining high detection accuracy.
(2) To meet the path planning requirements of robotic manipulators in rebar binding tasks, systematic improvements are introduced to the RRT-Connect algorithm in terms of sampling strategies, tree expansion, node reconnection, and path pruning, effectively improving planning efficiency and reducing path redundancy.

The remainder of this paper is organized as follows. Section 2 introduces the overall architecture and functional components of the rebar binding robot system. Section 3 presents the proposed rebar binding point recognition and localization method and the manipulator binding path planning algorithm in detail. Section 4 validates the effectiveness of the proposed methods through simulations and experiments, followed by analysis and discussion of the results. Finally, Section 5 concludes the paper and outlines directions for future research.

## 2. Overview of Rebar Binding Robot

**Figure 1:**
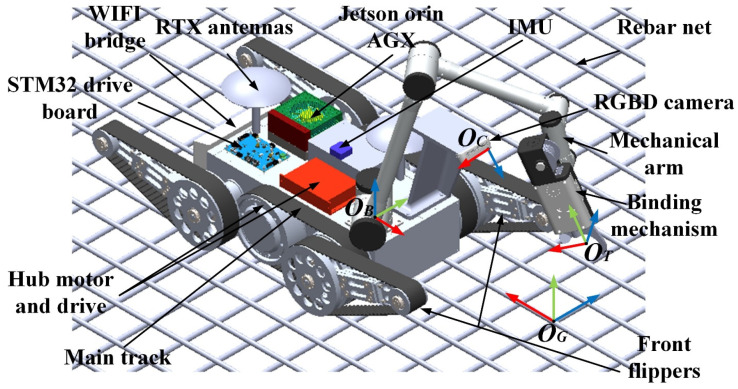
Overview of rebar binding robot.

As illustrated in Figure 1, the rebar binding robot system mainly consists of a locomotion subsystem and a binding execution subsystem. The locomotion subsystem adopts a tracked configuration, in which two main tracks are driven in a differential manner by hub motors, while the front and rear swing arms are independently actuated by servomotors to adjust the robot posture. The tracked locomotion mechanism offers advantages such as low power consumption, simple control strategy, and stable motion performance [38]. Compared with V-shaped wheel mechanisms that rely on rebar meshes as traveling rails, the tracked structure exhibits lower dependency on the rebar mesh layout and demonstrates stronger adaptability to complex construction environments. In addition, the introduction of front and rear swing arms enhances the robot’s obstacle-crossing capability, enabling complex motions such as autonomous climbing onto and descending from rebar meshes.

The binding execution subsystem consists of a six-degree-of-freedom articulated manipulator and a rebar binding tool. By coordinating their motions, the system is capable of automatically binding rebar intersection nodes located in front of the robot. Several coordinate frames involved in the system are defined in Figure 1: $O_{G}$ denotes the binding point coordinate frame, $O_{T}$ represents the rebar binding tool coordinate frame, $O_{C}$ corresponds to the camera coordinate frame, and $O_{B}$ indicates the manipulator base coordinate frame, where the red, green, and blue axes correspond to the x-, y-, and z-directions, respectively.

Based on prior work, the robot system has already achieved autonomous localization, mapping, and coverage path planning in rebar mesh environments. Using this rebar binding robot as the research platform, this paper focuses on the problems of binding point recognition and pose localization, as well as manipulator path planning for rebar binding tasks. Specifically, the study addresses the estimation of the pose of the binding point coordinate frame $O_{G}$ with respect to the manipulator base coordinate frame $O_{B}$, and the motion path planning of the manipulator from the rebar binding tool coordinate frame $O_{T}$ to the target binding point coordinate frame $O_{G}$.

## 3. Methods

This section focuses on the proposed rebar binding robot system and presents the methods for binding point recognition and pose localization, as well as the path planning and generation algorithms for robotic manipulator-based rebar binding operations.

### 3.1. Binding Point Recognition and Localization

The recognition and localization of rebar binding points are performed in two main stages. First, based on machine vision and deep learning techniques, the rebar binding points and their pose-defining keypoints are detected, and their three-dimensional spatial coordinates are obtained in the camera coordinate frame. Subsequently, using the hand–eye calibration results, the spatial coordinates of these keypoints are transformed from the camera coordinate frame to the manipulator base coordinate frame. Second, after acquiring the three-dimensional coordinates of all keypoints in the manipulator base frame, vector and matrix operations are employed to further compute the pose of the target rebar intersection with respect to the manipulator base coordinate frame. This provides accurate and reliable target pose information for subsequent manipulator path planning and rebar binding execution.

#### 3.1.1. Rebar Binding Point Recognition

**Figure 2:**
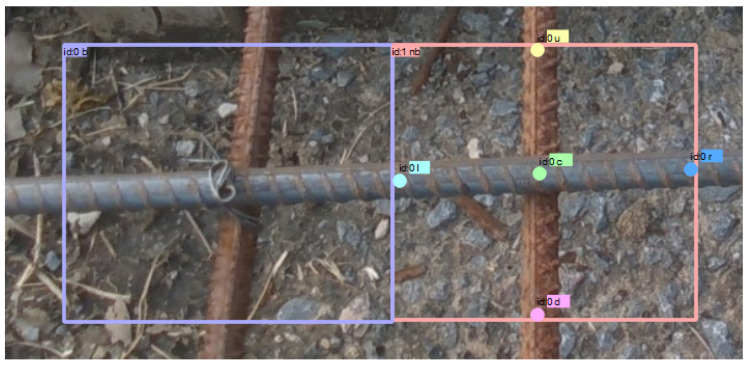
Rebar binding point recognition information.

To obtain the three-dimensional pose information of rebar binding points, the recognition results are defined as follows. As illustrated in Figure 2, the output includes the binding state of each rebar binding point, as well as the positions of five keypoints denoted as c, u, l, d, and r. These keypoints characterize the geometric structure of the rebar intersection region and provide essential spatial constraints for subsequent pose estimation of the target binding point.

Deep learning-based object detection methods exhibit strong robustness and good adaptability in complex environments. The YOLO family of algorithms [39], as representative single-stage object detectors, achieves favorable real-time performance while maintaining competitive detection accuracy. Among them, YOLOv8 is a mature version that integrates pose and keypoint detection capabilities. Its pose model can directly output target keypoints, which effectively meets the requirements of binding state determination and spatial pose recognition for rebar binding points. Therefore, the YOLOv8n-pose model is selected as the baseline model for rebar binding point recognition in this study. As the smallest-scale network in the YOLOv8 family, YOLOv8n-pose offers relatively low computational complexity and fast inference speed while preserving high detection accuracy. However, in the rebar binding application scenario—characterized by limited onboard computing resources and complex working environments—there remains room for further improvement in real-time performance. To address this issue, this paper introduces targeted improvements to the network structure and loss function of the YOLOv8n-pose model, as detailed below.

- Lightweight model improvement;

The Ghost module decomposes a standard convolution into two components: intrinsic feature generation via primary convolution and redundant feature expansion through linear transformations. By employing computationally efficient channel-wise linear operations to generate redundant feature maps, the Ghost module significantly reduces model parameters and computational complexity while preserving the output feature dimensions. Inspired by the design principle of the Ghost module, this paper constructs a multi-scale extended Ghost convolution module, termed Multi-Scale Ghost Convolution (MSGConv), to enhance multi-scale feature representation capability. Furthermore, C2fMSGR and MSGELAN structures based on the proposed MSGConv module are designed and employed to replace selected standard convolution layers and C2f modules in the YOLOv8n-pose network. This strategy enables effective model lightweighting while maintaining strong feature representation capability and improving overall computational efficiency.

The MSGConv module extends the design philosophy of GhostConv—namely, “primary feature generation plus lightweight convolutional expansion”—by introducing a parallel multi-scale branch structure to further enhance feature representation capability. Specifically, a standard convolution layer cv1 is first employed to project the input feature map x from $c_{1}$ channels to an intermediate channel dimension $c_{m}=c_{2}$/2, yielding the main-branch feature map $x_{1}$. Subsequently, $x_{1}$ is partitioned along the channel dimension into g sub-feature maps, denoted as $x_{2}=\left\{x_{2}^{\left(1\right)},x_{2}^{\left(2\right)},\ldots ,x_{2}^{\left(g\right)}\right\}$, where each sub-feature map contains $c_{m}/g$ channels. For different sub-feature maps, depthwise convolutions or group convolutions with kernel size $k_{s}$ are applied, thereby constructing multi-scale feature representations with diverse receptive fields. Finally, the main-branch feature $x_{1}$ and the outputs of all scale-specific branches are concatenated along the channel dimension and fused through a 1 × 1 convolution layer to restore the target output channel dimension $c_{2}$. By leveraging group and depthwise convolutions within a parallel multi-scale architecture, the proposed MSGConv module enhances feature representation capability while significantly reducing the number of parameters and computational complexity, thus preserving the lightweight nature of the module. The forward computation of MSGConv can be formally expressed as:

(1) $$x_{1}=cv1(x)$$

(2) $$x_{2}^{\left(i\right)}={Conv}_{k_{i}}\left(x_{1}^{\left(i\right)}\right),i=1,2,\ldots ,g$$

(3) $$\mathrm{out}={Conv}_{1\times 1}\left(concat\left(x_{1},x_{2}^{\left(1\right)},x_{2}^{\left(2\right)},\ldots ,x_{2}^{\left(g\right)}\right)\right)$$

Based on the above design, a C2fMSGR module is proposed, which integrates the information splitting and efficient feature aggregation mechanism of the C2f structure with multi-scale Ghost units equipped with residual connections (Multi-Scale Ghost with Residual, MSGA). Specifically, the input feature map is first projected to 2c channels via the convolution layer cv1 and then split along the channel dimension into two parts, denoted as $y_{0}$ and $y_{1}$, each containing c channels. Subsequently, $y_{1}$ is sequentially fed into n MSGA Bottleneck units to extract multi-scale features and generate multiple intermediate feature maps. Finally, $y_{0}$ and all intermediate features are concatenated along the channel dimension and fused through a 1 × 1 convolution layer cv2, producing an output feature map with $c_{2}$ channels. By inheriting the advantages of efficient information flow and residual feature fusion from the C2f structure while introducing multi-scale Ghost-based feature extraction, the proposed C2fMSGR module enhances feature representation capability under relatively low computational complexity.

In addition, an MSGELAN structure is designed for feature fusion. This structure first employs two parallel 1 × 1 convolution branches to project the input feature into a low-rank channel space, where branch cv1 includes an activation function and branch cv2 does not. Both branches output $c^{\prime }=c_{2}$/4 channels. Based on the output of branch cv1, an MSGBottleneck unit (denoted as m1) is introduced to expand the channel dimension to $2c^{\prime }$. Subsequently, the feature maps from cv1, cv2, and m1 are concatenated along the channel dimension and fused via a 1 × 1 convolution layer cv3 to generate the final output feature. By preserving multi-scale semantic information while effectively reducing computational complexity, the MSGELAN structure is particularly well suited for feature fusion in the Feature Pyramid Network (FPN) or Neck stage.

**Figure 3:**
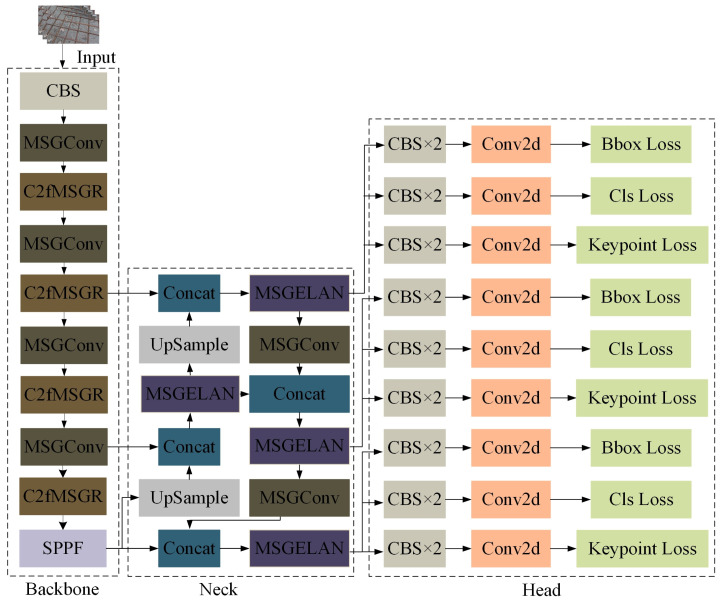
Network architecture of the lightweight YOLOv8n-pose model.

The overall network architecture of the lightweight YOLOv8n-pose model after the proposed modifications is illustrated in Figure 3.

- Loss function improvement;

After applying the lightweight structural optimization, the number of model parameters is reduced from 3.3 M to 1.97 M, and the computational complexity decreases from 8.3 GFLOPs to 5.7 GFLOPs. Moreover, real-time inference performance exceeding 48 FPS is achieved on the onboard Jetson AGX Orin platform (Nvidia Corporation, Santa Clara, CA, USA). Although the lightweight design significantly reduces computational overhead, it may also weaken the feature representation capability for keypoints to some extent, thereby imposing higher requirements on convergence stability and hard-sample learning. Consequently, it is necessary to introduce a more stable loss function that is more sensitive to hard samples to improve the overall convergence behavior of keypoint prediction.

The original YOLOv8 model employs the BCEWithLogitsLoss, which exhibits limited adaptability under complex background interference and dynamically changing sample distributions, making it difficult to effectively balance the learning of high-confidence samples and low-confidence hard samples during training. To address this issue, an adaptive threshold focal loss function is introduced in this paper. By dynamically adjusting the modulation factor, the proposed loss function enhances the model’s focus on hard samples and improves the convergence stability and robustness of keypoint prediction.

In the standard binary cross-entropy (BCE) loss, let the network output be $\hat{y}$, the predicted probability after the Sigmoid function be $p=\sigma (\hat{y})$, and the ground-truth label be y. The BCE loss is defined as:

(4) $$L_{BCE}(p,y)=-[ylog(p)+(1-y)log(1-p)]$$

Based on this formulation, the proposed loss function retains the BCE loss as the core term while introducing three key enhancements. First, to unify the confidence representation of positive and negative samples, a dynamic prediction confidence $p_{t}$ is defined as:

(5) $$p_{t}=yp+(1-y)(1-p)$$

where $p_{t}=p$ for positive samples and $p_{t}=1-p$ for negative samples, reflecting the model’s confidence in the current prediction. For each training batch, the mean confidence is computed as:

(6) $$\mu_{t}=\frac{1}{N}\sum_{i=1}^{N}p_{t,i}$$

And an exponential moving average is employed to update the dynamic threshold ${\bar{p}}_{t}=\lambda {\bar{p}}_{t-1}+(1-\lambda )\mu_{t}$.

This threshold characterizes the overall prediction difficulty of the network at the current training stage. Subsequently, an adaptive focal modulation factor γ is introduced and defined as:

(7) $$\gamma =-log\left({\bar{p}}_{t}\right)$$

As training progresses, when the model exhibits unstable predictions and higher sample difficulty (i.e., lower ${\bar{p}}_{t}$), the value of γ increases adaptively, thereby overcoming the limitation of a fixed γ that cannot accommodate different training phases. Finally, samples are partitioned according to their confidence levels, and different modulation strategies are applied. For high-confidence samples ($p_{t}>0.5$), the modulation term is defined as $M_{\mathrm{high}}={\left(1-p_{t}\right)}^{\gamma }$; for low-confidence samples ($p_{t}\leq 0.5$), the modulation term is defined as $M_{low}={\left(1.5-p_{t}\right)}^{-log\left(p_{t}\right)}$. When $p_{t}$ is small, the term $-log\left(p_{t}\right)$ becomes large, effectively enhancing hard-sample learning and alleviating gradient vanishing. The final modulation factor and the proposed loss function are given by:

(8) $$M=M_{high}+M_{low}$$

(9) $$L_{OPT}=M\cdot L_{BCE}$$

#### 3.1.2. Rebar Binding Point Localization

Using the proposed rebar binding point recognition model, the pixel coordinates of five keypoints corresponding to each binding point can be obtained in the image plane. Based on the camera imaging model, the three-dimensional coordinates of each keypoint in the camera coordinate frame $O_{C}$ can be computed through the following matrix transformation:

(10) $$\left[\begin{array}{c} x_{c}\\ y_{c}\\ z_{c} \end{array}\right]=z_{c}K^{-1}\left[\begin{array}{c} u\\ v\\ 1 \end{array}\right]$$

where (u,v) denotes the pixel coordinates of the keypoint in the image plane, $z_{c}$ is the depth value measured by the depth camera at the corresponding pixel, and K represents the camera intrinsic matrix, given by:

(11) $$K=\left[\begin{array}{ccc} f_{x} & 0 & c_{x}\\ 0 & f_{y} & c_{y}\\ 0 & 0 & 1 \end{array}\right]$$

After camera calibration and hand–eye calibration, the homogeneous transformation matrix **T*_CB_***, which describes the pose of the camera coordinate frame $O_{C}$ with respect to the manipulator base coordinate frame $O_{B}$, can be obtained. Accordingly, the keypoints can be transformed from the camera coordinate frame to the manipulator base coordinate frame as follows:

(12) $$\left[\begin{array}{c} x_{b}\\ y_{b}\\ z_{b}\\ 1 \end{array}\right]=T_{CB}\left[\begin{array}{c} x_{c}\\ y_{c}\\ z_{c}\\ 1 \end{array}\right]$$

**Figure 4:**
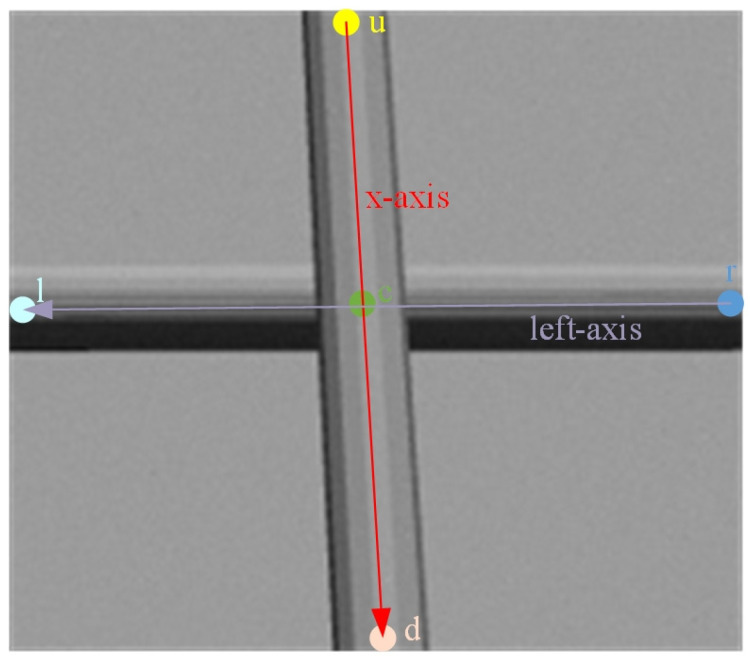
Construction of the target coordinate frame for the rebar binding point.

Since the five keypoints are not coplanar in three-dimensional space, a dedicated target coordinate frame $O_{G}$ is constructed to accurately describe the spatial pose of the rebar binding point, as illustrated in Figure 4. Specifically, the vector from keypoint u to d is defined as the x-axis direction, while the vector from keypoint r to l is defined as an auxiliary direction (referred to as the left axis). The positive direction of the y-axis is then obtained by the cross product of the x-axis and the left axis, and the positive direction of the z-axis is determined by the cross product of the x-axis and the y-axis. The keypoint c is selected as the origin of the coordinate frame, thereby fully defining the spatial pose of the target rebar binding point.

**Figure 5:**
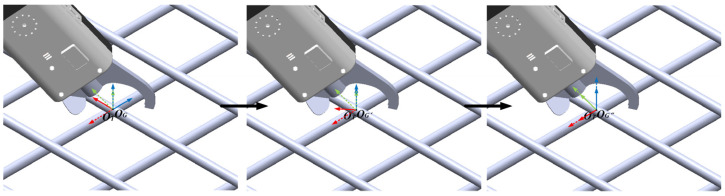
Construction of the target pose of the rebar binding tool.

On this basis, as illustrated in Figure 5, the target coordinate frame $O_{G}$ of the rebar binding point is first rotated by 45° about its y-axis to obtain an intermediate coordinate frame $O_{G}^{\prime }$. Subsequently, $O_{G}^{\prime }$ is rotated by −45° about its z-axis, resulting in the final coordinate frame $O_{G}^{\prime \prime }$. Based on $O_{G}^{\prime \prime }$, the target pose matrix of the binding tool, denoted as **T*_G_***, is constructed. This matrix represents the desired pose of the manipulator end-effector in the manipulator base coordinate frame. By controlling the manipulator to drive the binding tool to this target pose, the automatic binding operation at the corresponding rebar intersection point can be accomplished.

### 3.2. Manipulator Path Planning for Rebar Binding

After obtaining the target pose of the rebar binding tool, a collision-free motion path from the current pose to the target pose must be planned for the manipulator, while satisfying reachability and safety constraints and minimizing the path length as much as possible. The RRT family of algorithms, which is based on random sampling, is well suited for manipulator path planning in continuous high-dimensional spaces. However, conventional RRT methods often suffer from large path redundancy, suboptimal solutions, and relatively low search efficiency. The RRT-Connect algorithm [40] significantly improves planning efficiency by introducing bidirectional tree expansion and a fast connection strategy, and has become one of the most widely used classical algorithms for manipulator path planning.

Considering the characteristics of rebar mesh environments, where the free space is relatively large while obstacles are densely distributed around the target region, this paper proposes a series of improvements within the RRT-Connect framework. Specifically, enhancements are introduced from four aspects—sampling strategy, tree expansion scheme, node reconnection mechanism, and path pruning method—to further improve the overall efficiency and path quality of manipulator path planning for rebar binding tasks.

#### 3.2.1. Goal-Guided Adaptive Sampling Strategy

In rebar mesh environments, obstacles are mainly distributed on and around the rebar plane. From a spatial distribution perspective, obstacles are densely clustered in the vicinity of the target pose, whereas the region around the manipulator’s initial pose is relatively sparse and can be approximately regarded as free space. Conventional RRT-based algorithms typically adopt a uniform random sampling strategy over the entire configuration space. In obstacle-sparse regions, this strategy tends to generate a large number of redundant explorations, resulting in wasted computational resources; meanwhile, in obstacle-dense regions, it often produces numerous invalid samples that lead to collisions, thereby significantly increasing the cost of collision checking. Consequently, a single uniform sampling strategy is inadequate for environments with highly non-uniform obstacle distributions, such as rebar mesh scenarios.

To improve sampling efficiency and enhance the quality of random samples $Q_{rand}$, a goal-guided sampling strategy inspired by the Monte Carlo ε-Greedy principle is introduced in this paper. Specifically, with probability ε, the target pose $Q_{goal}$ is directly selected as the sampling point to strengthen guidance toward the goal region; otherwise, uniform random sampling is performed to preserve global exploration capability. The proposed goal-guided sampling policy is denoted as $\pi (Q_{rand})$, and its mathematical formulation is given in Equation (13).

(13) $$\pi (Q_{rand})=\left\{\begin{array}{r} 1-\frac{\varepsilon }{\left\|M\right\|}\left(\left\|M\right\|-1\right),\;Q_{init}=Q_{goal},\\ \frac{\varepsilon }{\left\|M\right\|},\;Q_{init}\neq Q_{goal}. \end{array}\right.$$

In Equation (14), $\left\|M\right\|$ denotes the size of the sampling space, i.e., the dimensionality of the manipulator configuration space. By introducing a goal-bias mechanism, the proposed sampling strategy $\pi (Q_{rand})$ assigns a higher probability to selecting the target configuration $Q_{goal}$, while ensuring that all other configurations retain non-zero sampling probabilities. This design enhances the search efficiency in the goal region while preserving exploration over the entire configuration space. Specifically, for any non-goal configuration, the sampling probability satisfies the following inequality:

(14) $$1-\frac{\varepsilon }{\left\|M\right\|}\left(\left\|M\right\|-1\right)=\frac{\varepsilon }{\left\|M\right\|}+1-\varepsilon \geq \frac{\varepsilon }{\left\|M\right\|}$$

This inequality indicates that a strictly positive lower bound is guaranteed for the sampling probability of any configuration, thereby theoretically ensuring global reachability and probabilistic completeness of the algorithm. The parameter ε∈[0,1] is used to balance exploration and goal-directed exploitation in the proposed sampling strategy.

#### 3.2.2. Distance-Decay-Based Variable Step-Size Expansion Strategy

In RRT-based algorithms, the expansion step size L is typically set to a fixed value, which directly affects both search efficiency and path quality. A large step size can accelerate the search process but may reduce path precision, whereas a small step size facilitates the generation of high-quality paths at the expense of search efficiency. To balance these conflicting requirements, a distance-decay-based adaptive step-size expansion strategy is proposed in this paper, in which the step size L decays exponentially with the distance between the nearest node $Q_{near}$ and the target node $Q_{goal}$. This strategy enables rapid exploration with larger step sizes in the early stage of the search, while automatically reducing the step size as the search approaches the target region, thereby improving planning accuracy. The step-size adaptation is defined as follows:

(15) $$L=L_{0}e^{-\left\|Q_{near}-Q_{goal}\right\|}$$

#### 3.2.3. Energy-Optimal Node Reconnection Strategy

The conventional RRT-Connect algorithm does not explicitly incorporate a path optimization mechanism, and the generated paths often exhibit high redundancy. To improve path quality, this paper draws inspiration from the node reconnection strategy of RRT*, where the parent node of a newly added node $Q_{new}$ is reselected during the connection process to achieve local path optimization.

Furthermore, since the joints of a robotic manipulator differ significantly in terms of structural dimensions, actuation capabilities, and motion costs, the path cost cannot be reasonably measured by a simple accumulation of joint angle variations. To address this issue, a weighted path cost function J is defined, in which different weights are assigned to individual joints to suppress excessive motion of large joints while encouraging fine adjustments of smaller joints. The cost function is formulated as follows:

(16) $$J=\sum_{k}\sum_{i=1}^{6}\omega_{i}{\left\|Q_{k}^{i}-Q_{parent}^{i}\right\|}^{2}$$

where $Q_{k}^{i}$ denotes the angle of the *i*-th joint at node k, $Q_{parent}^{i}$ represents the corresponding joint angle of its parent node, and $\omega_{i}$ is the weight coefficient associated with the *i*-th joint. By accumulating the weighted local costs between adjacent nodes and backtracking along the parent relationships, the total path cost from the current node $Q_{new}$ to the initial node $Q_{init}$ can be obtained, which serves as the criterion for node reconnection and path optimization.

#### 3.2.4. Path Optimization via Pruning and Reorganization

**Figure 6:**
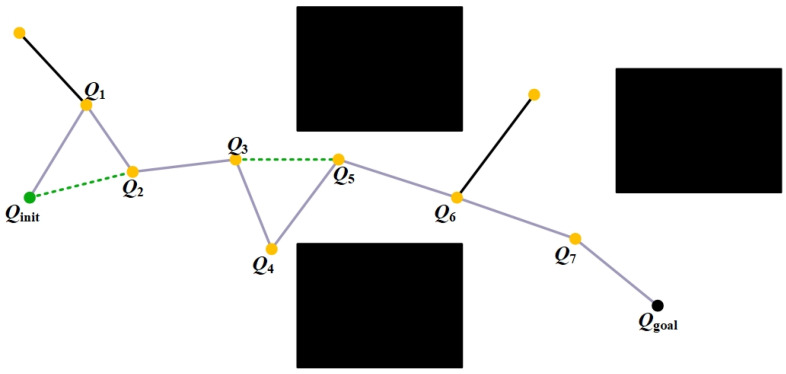
Illustration of the path pruning and reorganization strategy.

The feasible paths obtained from the search process often contain a large number of redundant nodes, which can significantly increase the computational burden of subsequent trajectory planning and execution. Therefore, it is necessary to perform pruning optimization on the initial planning results to improve path quality. To this end, this paper proposes a pruning and reorganization strategy that combines path cost evaluation with collision checking. As illustrated in Figure 6, the path cost J is first initialized to record the total cost of the current best path. The node sequence of the feasible path is then traversed, during which the path cost between nodes is evaluated and direct connections between non-adjacent nodes are attempted to construct shorter candidate paths. If the total cost of a candidate path is lower than the current optimal value, collision checking is performed for the proposed direct connection. Upon confirming that the connection is collision-free, the path node set is updated accordingly. Finally, an optimized pruned path is reconstructed based on the updated node sequence (shown as the green dashed line in Figure 6), thereby effectively reducing path redundancy while ensuring path feasibility and improving overall path quality.

## 4. Simulation and Experimental Evaluation

To verify the effectiveness of the proposed methods and algorithms, rebar binding point detection experiments and manipulator path planning simulation tests were conducted.

### 4.1. Binding Point Detection Experiments

A total of 2682 images of rebar meshes were collected at different time periods and under varying camera poses. The images include both unbound and bound rebar binding points, covering rebar surfaces in both rusted and non-rusted conditions. All images have a resolution of 1280 × 720. After data acquisition, all images were manually annotated to construct the rebar binding point detection dataset, as illustrated in Figure 2. Specifically, bounding boxes labeled as “b” represent bound rebar binding points, while those labeled as “nb” correspond to unbound binding points. In addition, five keypoints were annotated for each unbound binding point, including the center point “c”, upper point “u”, left point “l”, lower point “d”, and right point “r”.

To increase the dataset size, enhance model generalization capability, and reduce the risk of overfitting, various data augmentation techniques were applied to the original images, including rotation, scaling, cropping, noise injection, brightness adjustment, and mix-based augmentation. After augmentation, a total of 10,728 rebar mesh images were obtained. The dataset was then divided into training and validation sets according to a ratio of 9:1 for subsequent model training and performance evaluation. The hardware and software environment as well as the training parameter configurations are summarized in Table 1 and Table 2, respectively.

**Figure 7:**
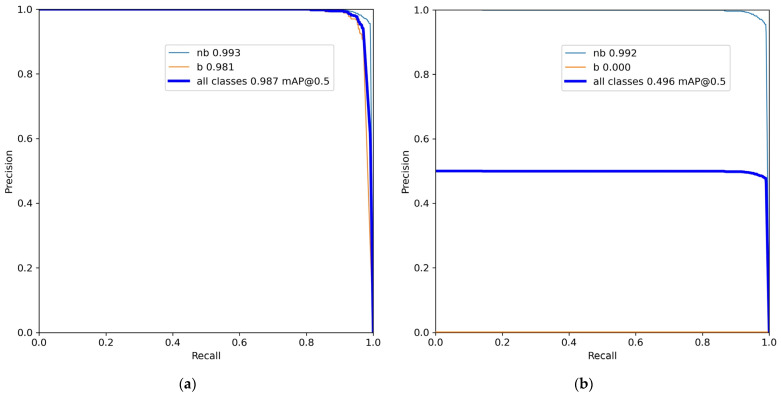
Precision–recall (P–R) curves of the YOLOv8n-pose-Binding model on the validation set: (**a**) P–R curve for bounding box detection; (**b**) P–R curve for keypoint detection.

The precision–recall (P–R) curves of the improved model (hereafter referred to as YOLOv8n-pose-Binding) on the validation set are illustrated in Figure 7. Specifically, Figure 7a presents the P–R curve for bounding box detection, while Figure 7b shows the P–R curve for keypoint detection.

As shown in Figure 7a, the proposed model demonstrates excellent bounding box detection performance on the validation set, achieving a Box mAP@50 of 0.987. This result indicates that the model can accurately identify the target regions of rebar binding points. Figure 7b depicts the P–R curve for keypoint detection. It should be noted that the “b” class corresponds to bound rebar binding points, for which keypoint detection is not required in practical applications. Moreover, keypoints for this class were not annotated during dataset construction; therefore, the corresponding P–R curve does not carry practical evaluation significance. The dark blue all classes curve represents the average performance across all categories. Since the keypoint-related values for the “b” class are zero, this curve effectively reflects half of the performance of the “nb” class. Overall, the proposed model achieves a Pose mAP@50 of 0.992 for the “nb” (unbound) class, indicating that YOLOv8n-pose-Binding also attains high accuracy and robustness in rebar binding point keypoint detection.

Table 3 presents a comprehensive performance comparison of the YOLOv8n-pose model before and after the proposed improvements on the validation and test sets. The results demonstrate that the combination of the lightweight network architecture and the adaptive threshold focal loss significantly reduces model complexity while maintaining or even improving keypoint detection accuracy. Specifically, the lightweight design reduces the number of parameters from 3.3 M to 1.97 M and decreases the computational cost (GFLOPs) from 8.3 to 5.7. Consequently, the inference speed on the Jetson AGX Orin platform increases from 34.2 FPS to 48.5 FPS, satisfying the real-time requirements of online rebar binding operations.

Although the simplified network structure leads to a slight decrease in the mAP@50–95 for bounding box detection, this degradation is effectively compensated by the enhanced hard-sample learning capability introduced by the adaptive threshold focal loss. As a result, the Pose detection mAP@50–95 improves from 0.987 to 0.992, indicating enhanced keypoint localization accuracy and convergence stability. These results verify that the proposed MSGA-based multi-scale feature enhancement and the dynamic hard-sample modulation of the adaptive threshold focal loss can effectively improve keypoint detection performance while reducing computational overhead. Overall, the YOLOv8n-pose-Binding model achieves a more favorable balance among accuracy, inference speed, and resource consumption, making it particularly suitable for deployment in resource-constrained industrial rebar binding robot scenarios.

### 4.2. Simulation Evaluation of the Improved RRT-Connect Algorithm

To verify the effectiveness and superiority of the proposed improved RRT-Connect path planning algorithm, ablation experiments were first conducted to analyze the contribution of each algorithmic enhancement. Subsequently, path planning simulation experiments were carried out in both two-dimensional planar environments and three-dimensional spatial environments. Comparative analyses were performed against the RRT, RRT*, and conventional RRT-Connect algorithms. All algorithms were executed under identical environment models and constraint conditions to ensure the fairness and reproducibility of the comparison results. The main parameter settings of the improved RRT-Connect algorithm are summarized in Table 4.

**Figure 8:**
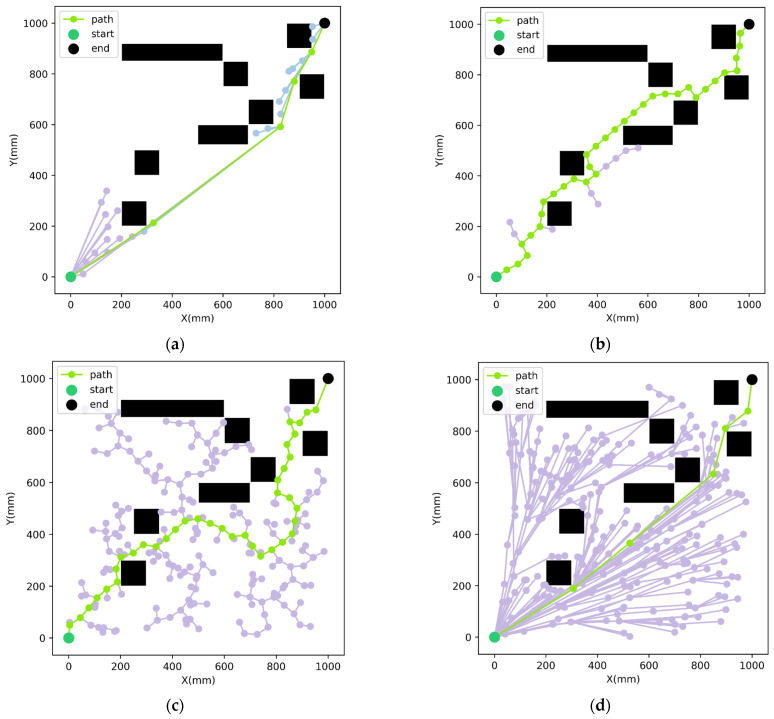
Path planning results in the two-dimensional planar environment: (**a**) Improved RRT-Connect algorithm; (**b**) RRT-Connect algorithm; (**c**) RRT algorithm; (**d**) RRT* algorithm. Note: Purple represents the search path of the unidirectional tree, and blue represents the search path at the other end of the bidirectional tree.

First, ablation experiments were conducted in the two-dimensional planar environment shown in Figure 8a to evaluate the individual contributions of each proposed improvement strategy. The search space was defined as $M=[\left[0,1000\right],\left[0,1000\right]]$, with the start point set to $\left[0,0\right]$ and the goal point set to $\left[1000,1000\right]$, both in millimeters. Eight obstacles were generated within the environment. The four improvement modules were enabled progressively to construct comparative configurations using a step-by-step addition strategy. To objectively evaluate the performance of each configuration, ten independent trials were conducted for each case, and the average results are reported in Table 5.

The results in Table 5 indicate that each module contributes positively to the overall performance of the algorithm at different levels. After introducing the goal-guided adaptive sampling strategy, the algorithm enhances its search bias toward the target region while preserving global exploration capability, enabling the random trees to expand more efficiently toward the goal. As a result, both the path length and the number of path nodes are reduced. By further incorporating the distance-decay-based variable step-size expansion strategy, the tree expansion speed is accelerated during the early search stage, while higher expansion precision is achieved when approaching the target region, thereby effectively reducing invalid expansions and redundant nodes. Subsequently, the energy-weighted node reconnection mechanism improves path smoothness by constraining motion costs through joint-weighted optimization, leading to a significant reduction in the number of path nodes and an overall improvement in path quality. Finally, the path pruning and restructuring strategy compresses redundant nodes under collision-free constraints, further simplifying the path structure. Overall, the proposed enhancement modules exhibit complementary effects in terms of path quality improvement and node reduction, resulting in superior comprehensive planning performance.

To further compare the performance of the proposed improved RRT-Connect path planning algorithm with other commonly used algorithms, path planning simulation tests were conducted in the same two-dimensional planar environment with identical start and goal configurations. The path planning results of each algorithm are illustrated in Figure 8, and the corresponding quantitative comparison results are summarized in Table 6.

As illustrated in Figure 8, the proposed improved RRT-Connect algorithm outperforms the comparative algorithms in terms of overall path quality. Specifically, the conventional RRT algorithm produces the longest paths with the largest number of redundant nodes, resulting in the poorest path quality. Although RRT* is capable of generating relatively optimal paths, it suffers from significantly longer planning times and lower planning efficiency. Considering the randomness inherent in sampling-based planning, 10 independent trials were conducted for each algorithm and the average results were reported to ensure objective performance evaluation, as summarized in Table 6.

The results in Table 6 indicate that the proposed improved RRT-Connect algorithm achieves a shorter average path length while significantly reducing the number of path nodes. Compared with RRT and conventional RRT-Connect, the generated paths are more compact and smoother, leading to higher overall path quality. Compared with RRT*, the proposed method substantially reduces the planning time while maintaining comparable path quality, demonstrating a clear advantage in overall performance.

**Figure 9:**
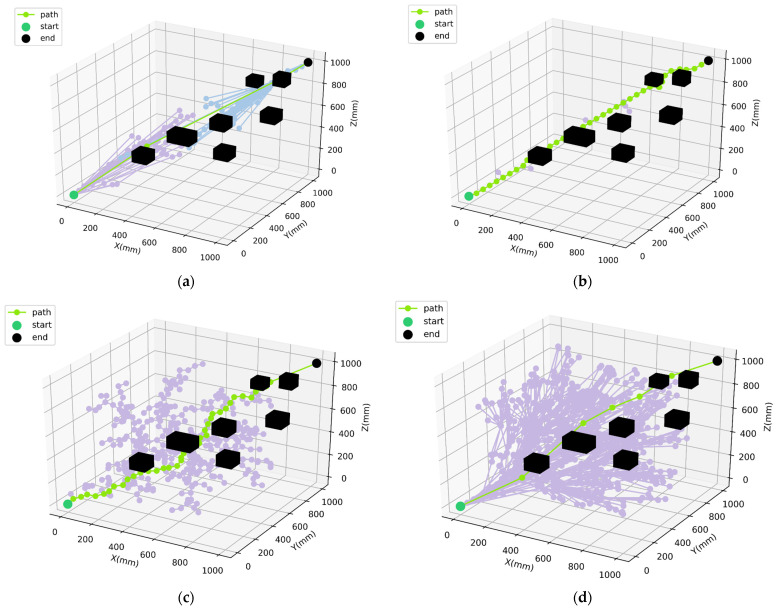
Path planning results in the three-dimensional spatial environment: (**a**) Improved RRT-Connect algorithm; (**b**) RRT-Connect algorithm; (**c**) RRT algorithm; (**d**) RRT* algorithm. Note: Purple represents the search path of the unidirectional tree, and blue represents the search path at the other end of the bidirectional tree.

To further validate the generality and high-dimensional adaptability of the proposed algorithm, path planning simulation experiments for all compared algorithms were also conducted in a three-dimensional spatial environment. The 3D search space was defined as $M=[\left[0,1000\right],\left[0,1000\right],\left[0,1000\right]]$, with the start configuration at $\left[0,0,0\right]$ and the goal configuration at $\left[1000,1000,1000\right]$. Seven obstacles were generated within the workspace. The path planning results of different algorithms in the 3D environment are illustrated in Figure 9, and the corresponding quantitative comparisons are presented in Table 7.

As shown in Figure 9, the proposed improved RRT-Connect algorithm also exhibits superior path quality compared with the baseline methods in the three-dimensional environment. To mitigate the influence of randomness inherent in sampling-based planning, each algorithm was evaluated over 10 independent trials and the average results are reported in Table 7.

The results in Table 6 indicate that the improved RRT-Connect algorithm is capable of generating shorter and smoother paths in high-dimensional 3D spaces while significantly reducing the number of path nodes. Compared with RRT and conventional RRT-Connect, the proposed method effectively avoids the generation of excessive redundant nodes. Compared with RRT*, it achieves substantially higher planning efficiency while maintaining comparable or better path quality. These results clearly demonstrate the superior overall performance of the proposed improved RRT-Connect algorithm for path planning in high-dimensional spaces.

**Figure 10:**
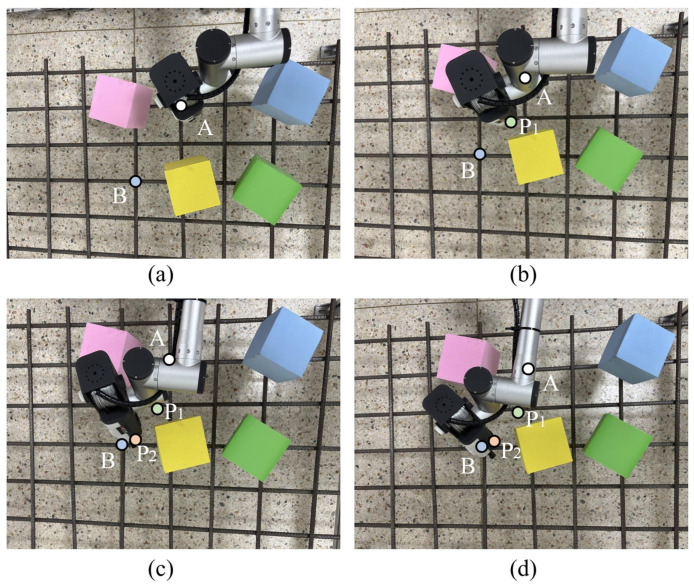
Manipulator path planning experiment.

In addition, a preliminary validation of the proposed improved RRT-Connect algorithm was conducted in real robotic manipulator path planning tasks. As shown in Figure 10, four static obstacles were arranged on the rebar plane, and the manipulator was commanded to move from the pose A corresponding to the completion of the previous binding operation to the target pose B associated with the next rebar intersection point. The initial pose, target pose, and environmental obstacle information were provided as inputs to the improved RRT-Connect algorithm. The intermediate waypoints along the planned path were labeled sequentially as P_1_, P_2_, and so on, according to their order along the trajectory.

As illustrated in Figure 10, the manipulator starts from the initial pose A, follows the planned trajectory through the intermediate waypoints P_1_ and P_2_, and finally reaches the target pose B smoothly. Throughout the motion process, collision-free constraints are strictly satisfied, and the static obstacles on the rebar plane are successfully avoided. These results demonstrate the feasibility and effectiveness of the proposed algorithm for real robotic manipulator path planning tasks.

Furthermore, under the same obstacle configuration and task settings, comparative experiments were conducted using the improved RRT-Connect, conventional RRT-Connect, RRT, and RRT* algorithms to evaluate their practical performance. Considering the inherent randomness of RRT-based methods, each algorithm was executed independently ten times. The average path length, computation time, and number of path nodes were recorded, and the overall comparison results are summarized in Table 8.

From Table 8, it can be observed that the improved RRT-Connect algorithm achieves the best performance in terms of average path length and average number of path nodes, resulting in more compact and concise planned paths. In terms of solution time, the proposed algorithm exhibits a slight increase compared with the baseline RRT-Connect algorithm. This is mainly attributed to the introduction of node reconnection and path pruning mechanisms, which increase the computational complexity of a single planning process. Nevertheless, the resulting planning time remains within an acceptable range for offline manipulator path planning tasks.

By further considering the standard deviations obtained from multiple repeated experiments as reported in Table 9, it can be seen that the improved RRT-Connect algorithm exhibits relatively small variations in key performance metrics, including path length, planning time, and number of path nodes. This indicates that the proposed algorithm maintains good stability and repeatability under the inherent randomness of sampling-based planning. Overall, the improved RRT-Connect algorithm demonstrates clear advantages in terms of path quality and engineering applicability, and can be effectively applied to practical path planning tasks for rebar binding manipulators.

## 5. Conclusions

This paper addresses the automation requirements of rebar binding robots operating in complex construction environments and investigates rebar binding-point recognition and localization methods as well as manipulator binding path planning strategies from a system integration and engineering implementation perspective. To satisfy the practical demands for real-time performance and stability on resource-constrained mobile platforms, a lightweight rebar binding-point recognition and localization model, termed YOLOv8n-pose-Binding, is proposed. By incorporating multi-scale Ghost convolution structures and designing an adaptive threshold focal loss within the YOLOv8n-pose framework, the proposed model reduces parameter count and computational complexity while achieving stable perception of key binding-point features, thereby meeting the requirements for online deployment on embedded platforms.

Based on the depth camera imaging model and hand–eye calibration results, a target coordinate system for rebar binding points is constructed, enabling accurate pose estimation of binding points in the manipulator base coordinate frame and providing reliable target pose inputs for automated binding operations. In terms of path planning, considering the uneven distribution of free space and obstacles in rebar mesh environments as well as the high-dimensional motion characteristics of robotic manipulators, a series of engineering-oriented improvements—including goal-guided sampling, variable step-size expansion, energy-weighted node reconnection, and path pruning—are introduced within the RRT-Connect framework. These strategies effectively enhance planning efficiency and reduce path redundancy.

Simulation and experimental results demonstrate that the proposed method outperforms the baseline approaches in both path quality and computational efficiency, providing an engineering reference for achieving stable and efficient automated operation of rebar binding robots.

Future work will focus on further expanding the dataset for rebar binding-point recognition and localization under more complex working conditions and conducting on-site experimental validation of rebar binding tasks. On this basis, the proposed methods will be jointly integrated with the robot’s localization, mapping, and navigation system to perform comprehensive system-level testing, ultimately enabling fully autonomous rebar binding operations in complex construction scenarios and further improving the overall robustness and intelligence of the system.

## Figures and Tables

**Table 1:** Hardware and software environment for model training.

Hardware Configuration		Software Configuration	
CPU	AMD 5995WX	Operating System	Ubuntu 22.04
		Python	3.8.13
GPU	24 GB NVIDIA RTX 4090	Pytorch	2.1.1
		Torchvision	0.16.1
		CUDA	12.1
Memory	128 GB DDR4	cuDNN	8.9.0.2

**Table 2:** Parameter settings for model training.

Parameter	Epochs	Batch	Patience	Optimizer	lr0	lrf	Weight_Decay	Seed
Value	400	32	100	AdamW	0.001	0.01	0.0005	0

**Table 3:** Performance comparison of the YOLOv8n-pose model before and after improvement on the validation sets.

Model	Box/Pose	Precision	Recall	mAP50	mAP50-95	GFLOPs	FPS ^1^
YOLOv8n-pose	Box	0.963	0.973	0.983	0.924	8.3	34.2
	Pose	0.972	0.983	0.987	0.987		
YOLOv8n-pose-Binding	Box	0.967	0.963	0.987	0.917	5.7	48.5
	Pose	0.968	0.98	0.992	0.992		

^1^ Since the models are deployed on a robot-mounted Jetson AGX Orin platform, all FPS results reported in this table are measured on the same platform.

**Table 4:** Parameter settings of the improved RRT-Connect algorithm.

Parameter	Value
ε	0.3
$L_{0}$	50
$\omega_{i}$	[1.0, 0.9, 0.8, 0.4, 0.3, 0.2]

**Table 5:** Ablation experiment results in the two-dimensional environment.

Group	Algorithmic Enhancement	Average Path Length (mm)	Average Planning Time (s)	Average Number of Path Nodes
B0	RRT-Connect	1710.57	0.0753	33.9
B1	B0 + goal-guided sampling	1658.40	0.1825	29.6
B2	B1 + variable step-size expansion	1589.70	0.5584	21.3
B3	B2 + energy-weighted node reconnection	1508.60	0.9621	12.1
B4	B3 + path pruning	1480.82	1.3977	5.8

**Table 6:** Quantitative comparison of path planning performance in the two-dimensional planar environment.

Algorithm	Improved RRT-Connect	RRT-Connect	RRT	RRT*
Average path length (mm)	1480.82	1710.57	1783.56	1471.29
Average planning time (s)	1.3977	0.0753	0.6399	2.8099
Average number of path nodes	5.8	33.9	35.5	8.4

**Table 7:** Quantitative comparison of path planning performance in the three-dimensional environment.

Algorithm	Improved RRT-Connect	RRT-Connect	RRT	RRT*
Average path length (mm)	1761.47	1959.57	2271.39	1787.27
Average planning time (s)	2.6186	0.0748	0.7102	2.9430
Average number of path nodes	4.4	41.8	39.5	5.7

**Table 8:** Comprehensive performance comparison of path planning algorithms.

Algorithm	Improved RRT-Connect	RRT-Connect	RRT	RRT*
Average path length (mm)	231.5	242.6	287.6	231.8
Average planning time (s)	2.28	0.45	0.98	8.22
Average number of path nodes	4.2	9.8	19.4	6.2

**Table 9:** Standard deviations of key performance metrics of the improved RRT-Connect algorithm over multiple trials.

Algorithm	Path Length (mm)	Planning Time (s)	Number of Path Nodes
Improved RRT-Connect	1.94	0.91	0.53

## Data Availability

The data presented in this study are available on request from the corresponding author.
